# Best practice versus actual practice: an audit of survey pretesting practices reported in a sample of medical education journals

**DOI:** 10.1080/10872981.2019.1673596

**Published:** 2019-10-31

**Authors:** Colleen Y. Colbert, Judith C. French, Alejandro C. Arroliga, S. Beth Bierer

**Affiliations:** a Education Institute, Cleveland Clinic and Cleveland Clinic Lerner College of Medicine of Case Western Reserve University, Cleveland, Ohio; b General Surgery Residency Program, Cleveland Clinic and Cleveland Clinic Lerner College of Medicine of Case Western University, Cleveland, Ohio; c Department of Medicine, Baylor Scott & White Health/Texas A&M HSC College of Medicine, Temple, Texas

**Keywords:** Audit, survey methodology, pretesting, questionnaire, questionnaire methodology, survey validation, healthcare

## Abstract

**Background**: Despite recommendations from survey scientists, surveys appear to be utilized in medical education without the critical step of pretesting prior to survey launch. Pretesting helps ensure respondents understand questions as survey developers intended and that items and response options are relevant to respondents and adequately address constructs, topics, issues or problems. While psychometric testing is important in assessing aspects of question quality and item performance, it cannot discern how respondents, based upon their lived experiences, interpret the questions we pose.

**Aim**: This audit study explored whether authors of medical education journal articles within audited journals reported pretesting survey instruments during survey development, as recommended by survey scientists and established guidelines/standards for survey instrument development.

**Methods**: Five national and international medical education journals publishing survey articles from Jan. 2014 – Dec. 2015 were audited to determine whether authors reported pretesting during survey development. All abstracts within all issues of these journals were initially reviewed. Two hundred fifty-one articles met inclusion criteria using a protocol piloted and revised prior to use.

**Results**: The number of survey articles published per journal ranged from 11 to 106. Of 251 audited articles, 181 (72.11%) described using a new instrument without pretesting, while 17 (6.77%) described using a new instrument where items were pretested. Fifty-three (21.12%) articles described using pre-existing instruments; of these, no articles (0%) reported pretesting existing survey instruments prior to use.

**Conclusions**: Findings from this audit study indicate that reported survey pretesting appears to be lower than that reported in healthcare journals. This is concerning, as results of survey studies and evaluation projects are used to inform educational practices, guide future research, and influence policy and program development. Findings apply to both survey developers and faculty across a range of fields, including evaluation and medical education research.

## Introduction

Despite recommendations from survey scientists and medical educators to assess the quality of survey items prior to use [1–24], survey instruments are frequently utilized in the field of medical education without a key step in question development: pretesting. For some medical education researchers, program evaluators, and curriculum designers, the omission of pretesting methodology (Table 1) may represent a gap in knowledge regarding question design [10]. For others, the time required for pretesting (i.e., assessing survey items prior to use to enhance construct validity and minimize measurement error) may seem unnecessary [5,14,25], especially when journals do not uniformly require descriptions of survey development [3,5,15]. One analysis of healthcare-related journals found that descriptions of question development (including assessment of question quality) were included in less than a quarter of studies reviewed [5]. In another analysis, only 36% of survey articles within a sample of critical care medicine journals reported pretesting methods or piloting of the instrument [26]. In a recent study focusing on surveys in research articles, Artino et al. [3] examined the overall quality of self-administered research survey instruments published in three high-impact factor health professions education journals in 2013 and found low rates of cognitive interviewing/testing, one type of pretesting, in their sample of 36 survey research articles.

**Table 1.** Definitions and descriptions.

Term	Definition
**Definitions**	
Pretesting	Pretesting, which occurs prior to pilot testing, refers to a variety of methods designed to assess survey quality and the question-response process (*see descriptions below*) during survey development and prior to data collection in a study, evaluation, or other project [2,10,15]. Unlike pilot testing, pretesting does not involve assessment of all aspects of survey administration. Pretesting methods include, but are not limited to: cognitive interviewing/testing, focus groups, behavioral coding, expert panels, and questionnaire appraisal tools. Methods often focus on sources and mitigation of response error, though newer approaches also explore how questions are perceived and interpreted across cultures, language groups, and other sub-populations [43].
Field testing or field trials	Field testing allows researchers to assess aspects of survey administration in the field, prior to piloting and survey launch [6]. Pretesting and field testing are sometimes combined (i.e., respondent time to completion may be captured during pretesting procedures). Researchers may focus on a portion of the administration, such as the ability of interviewers’ to make contact with subjects at home addresses or navigation problems in online surveys [49]. Field tests are critical when conducting research in remote locations in order to determine the feasibility of procedures and may involve study personnel only.
Pilot testing	Pilot testing occurs *after* pretesting the survey instrument and is focused on *overall* survey refinement. Where pretesting typically involves small numbers of respondents (e.g., one-on-one interviews in a laboratory or feedback from a small number of experts), piloting occurs under realistic conditions [6,27] with a larger sample of respondents from the target population. All survey procedures are examined. Piloting can help researchers determine which survey items should be dropped and which retained, and whether a study is feasible and worthwhile [27].
**Descriptions of Pretesting Methods**	
Cognitive testing or interviews	Cognitive testing or interviewing refers to a range of methods used to probe individual cognitive response processes and relies on capturing feedback about item comprehensibility from respondents who are similar to one’s target population [9,13,19,21–23,25,43]. These in-depth, one-on-one interviews are often conducted on a small scale (e.g., 10–30 respondents) [43], but valuable information can be gathered from even 5–6 respondents [23]. Cognitive testing/interviewing includes techniques such as *think a louds*, where respondents articulate their thought processes as they answer survey questions [9,13,21,28,43]. As this can be problematic for many respondents, retrospective verbal probing is now recommended by many researchers and involves respondent reflection after the fact regarding their response processes [22,29,43].
Focus groups	Focus groups are used across a range of disciplines, including, but not limited to, educational, sociological and healthcare research, business/marketing, and political science. Focus groups are used to explore how people think or feel about an issue through guided group discussion. In research studies, participants are typically selected based upon shared characteristics as they relate to the study topic. See Krueger & Casey [30]. While focus groups are often used to develop concepts for questionnaires [2,4], they can also be used when pretesting to assess question comprehension, response format, and even content coverage [2,19,31,32].
Behavioral Coding	Behavioral coding is primarily used with interviewer-led surveys and is a systematic method for exploring both interviewer and respondent behaviors in live or telephone interviews [12]. While it can be used to monitor behaviors to improve interview quality, it is also helpful in examining the behaviors of respondents (e.g., how many times respondents ask for clarification). Trained coders are used to examine videotapes or audio recording and apply codes to specific behaviors.
Expert feedback and expert panels	Feedback from individual experts or expert panels on content and questionnaire design is frequently used to ensure that appropriate content is covered by items [6,10,20]. This in turn can help to ensure that construct irrelevant variance is reduced [61].Depending upon the purpose and topic of the survey instrument, this feedback may be essential, but is not adequate in determining if questions will be understandable to specific target populations [6,9,13,21].
Survey or questionnaire appraisal tools	A type of standardized evaluation form, the questionnaire appraisal tool is completed by respondents and/or experts who typically first take the survey and then use the appraisal tool to evaluate item and instrument quality, providing information on item specificity, clarity and whether questions are leading [16,28,31,33]. Use of the appraisal tool can be followed by an in-person debriefing [31], where respondents are asked to elaborate on any items they found to be problematic (e.g., double-barreled, leading, etc.).
Other methods	For other techniques common in pretesting interviewer-led surveys (e.g., behavior coding of interview transcripts), see Blake [39], ASA [2], and Dillman et al. [10].

In this article, we describe the use of audit methodology to examine pretesting practices reported in five medical education journals, with the goal of improving the quality of survey question design and survey reporting practices. Audits compare current practices with best practices or guidelines/standards, with a goal of practice improvement [34–38]. During our audit, we examined medical education journal articles reporting a broad range of survey usage (e.g., survey instruments used in medical education research, program evaluation, curriculum development, innovations, etc.), and then compared these descriptions of survey development with recommendations from methodologists and scientists within the field of survey science [1,2,6,8–15,18,19,21,22,24,39]. Findings reported in this paper should be useful to survey developers across a range of fields, including program evaluation, and ensure the implementation of best practices.

### Background

Survey methodology includes the use of in-person, phone, or online interviews and self-administered questionnaires (SAQs) (e.g., paper, emailed, online), where a sample of respondents drawn from a target population is queried systematically with standardized instruments to examine aspects of the larger population [7,12,14,22]. The goal is for respondents to ‘interpret the questions consistently and as the question developer intended.’ (p. 2) [40]. Survey developers often assume that instruments will collect ‘true’ or accurate data [8,41]. Yet, this may not be the case, due to issues which arise during the question-response process [9,11,22,23].

## Question response processes

While some survey developers may view questions as ‘objective’ if they query for quantitative answers and ‘subjective’ if they query for narrative responses, no question is truly objective due to the array of factors which can prevent a survey developer’s intended meaning from being accurately communicated to respondents [2,6,9,10,12,18,19,42,43]. A question-response model, based in cognitive psychology, is often used to describe the question-response process [13,19,21,43]. According to this model, when respondents answer survey questions they engage in a complex series of iterative cognitive processes. These processes include question comprehension (from a literal and pragmatic standpoint); retrieval of relevant information; calculation, estimation or construction of required information; decisions of whether to tell the truth, how much to answer, and estimation of harm (i.e., judgment); and selection or formulation of a response, either by mapping answers to response options provided or answering open-ended questions [9,12,18,19,43,44]. As Collins [9] and Dillman et al. [10] have noted, respondents must engage in at least two additional steps with self-administered questionnaires: understanding that they are being asked to fill out a questionnaire and then understanding how to navigate through a survey tool on their own. Every juncture in this set of iterative processes provides potential sources of measurement error during data collection [9,13,44].

Approaches to answering questions also mirror conversational behaviors and are influenced by socio-cultural expectations and norms [13,18,19,42,45], which in turn affect how respondents approach the overall survey response process [44]. Ambiguous items are problematic for respondents and survey developers, as respondents may create their own meanings for unclear questions or ignore ambiguous items entirely. Additionally, meanings can vary across individuals, social groups, and cultures [9,12,44–46]. All of these factors may contribute to increased measurement error, thereby impacting score reliability, when surveys are fielded [6,19,45]. Even seemingly straightforward questions such as, ‘How much exercise do you get, on average?’ can generate responses rife with measurement error when a time period is omitted [6] or respondents are forced to calculate in order to select a response. At times, respondents may find the number of response options to be inadequate or inappropriate, thereby inserting measurement error into the data collection process. In other cases, respondents may be concerned about confidentiality of the response process (e.g., surveys concerning the workplace) and may skip questions or produce superficial or socially acceptable answers [9,12,42,46]. For SAQs, where respondents lack verbal cues and interviewer clarification, answering even simple questions can be problematic [9,12,18,19,44].

## Rationale for assessing survey questions

Validity involves making judgments about the extent to which sources of evidence support the adequacy and appropriateness of score interpretations and actions based upon those scores [47]. Examining response processes during pretesting provides an important source of validity evidence, as the validity of survey results is determined by multiple factors, including the comprehensibility of questions asked, adequacy of response options, overall survey design, and acceptability of the survey tool [8–10,18,25,41,48].

When respondents uniformly comprehend an item differently than survey developers intended, measurement error is introduced into survey data [2,9–13,21–23,44]. Even the most common terms (e.g., ‘child/children’ and ‘you’) used in survey questions have been interpreted in different ways by respondents [12,19]. Online, self-administered questionnaires can be especially challenging for some respondents due to visual design elements and navigational factors which can affect item completion and item comprehension [2,10,22]. Location (spacing/alignment) of visual design elements affects respondents’ perceptions of item relatedness and may cause respondents to perceive items as being related when they are not [10], which can affect the accuracy of responses.

While measurement error associated with survey use cannot be avoided [19,22,41,44], it can be minimized by following recommended steps during survey development [1,2,12,22], including assessing the quality of survey items prior to use [1,2,6,9,13,15,20,22,24]. Item assessment includes both quantitative and qualitative methods. Quantitative methods can help us to determine how widespread a problem is and how individual survey items perform compared with other items. Yet, these methods cannot tell us *why* an item is problematic, especially across socio-cultural groups. The acceptance of pretesting as a fundamental step in establishing survey item quality has prompted numerous government agencies to use cognitive interviews, one method of pretesting, to gather feedback to determine whether survey instruments need to be modified prior to use [44]. Pretesting not only helps to ensure respondents understand questions as survey developers intended, but also ensures that constructs, topics, issues, or problems are relevant and adequately represented from the point of view of survey participants. Miller noted that “Today there is little debate that question design – how questions are worded and the placement of those questions within the questionnaire – impacts responses.” (p. 2) [13].

## Pretesting methods

Pretesting refers to a variety of methods (e.g., cognitive interviews, focus groups, use of questionnaire appraisal tools, etc.) which are used to assess both questions and survey tool format prior to use [2,10,15]. Pretesting occurs *prior to* pilot testing, and most pretesting methods are participatory (i.e., respondents are made aware of their roles in assessing survey items) [15]. A hybrid approach, combining a variety of pretesting methods, can overcome limitations of any one method [2,9,10,22,39]. See Table 1 for descriptions of pretesting methods. Of note, while expert feedback is often important during the question design process, it cannot provide information on whether questions will be understandable (literally and pragmatically) or acceptable to members of specific target populations (e.g., patients, specific ethnic or cultural groups, etc.) due to differences in education, language, health literacy, reading level, and sociocultural factors between experts and survey respondents [2,6,9,13,19,44]. Content experts are typically not knowledgeable about differences in item functioning across cultures and languages, which is especially critical when survey tools are translated [44]. Determining how representative members of the target population will interact with and comprehend survey items is therefore considered to be a fundamental step in survey development [9,10,13,22–24,44]. Last, assessing the performance of a survey in the field and under realistic conditions (field testing) [1,49] is often combined with pretesting of online and self-administered surveys prior to pilot testing (Table 1).

## Purpose

The purpose of this study was to determine whether authors of medical education journal articles featuring survey methodology reported pretesting survey instruments during survey development, a best practice recommended by survey scientists and one of the established guidelines/standards for survey development [see 10,12,15].

### Methods

#### Review of the literature

Rather than rely solely on recommendations published within our own field, we adopted a transdisciplinary approach and reviewed literature from experts in the field of survey methodology/survey science [6,8,10–12,15,18,19,21–25,42,43,46,48] to identify best practices regarding pretesting methods.

#### Review of national/international guidelines

In keeping with audit methodology, we reviewed guidelines, best practices, and standards (grey literature) from the American Statistical Association’s Section on Survey Methods [2], Pew Research Center [14], the Statistical and Science Policy Office’s Subcommittee on Questionnaire Evaluation Methods [40], and the American Association of Public Opinion Research (AAPOR) [1]. AAPOR is the largest survey and public opinion research organization in the USA, whose members include employees of national and international governmental bodies (e.g., U.S. Census Bureau), academic/scientific institutions (e.g., NORC at the University of Chicago), private enterprise survey practitioners, and survey scientists from all over the world.

**Figure 1.**
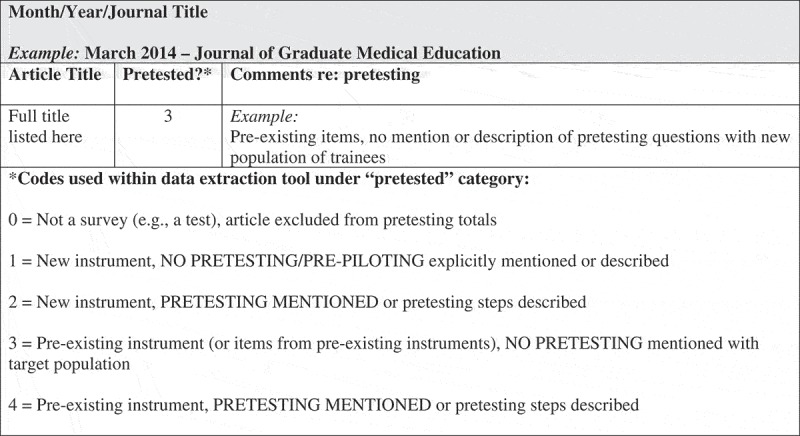
Data extraction form sample.

In addition to offering guidelines concerning overall survey quality (Figure 1), AAPOR offers specific guidance concerning pretesting in its Best Practices for Survey Research:

5. Take great care in matching question wording to the concepts being measured and the population studied:

“ … Ways should be devised to keep respondent mistakes and biases (e.g., memory of past events) to a minimum, and to measure those that cannot be eliminated. To accomplish these objectives, well-established cognitive research methods (e.g., paraphrasing and “think-aloud” interviews) and similar methods (e.g., behavioral coding of interviewer-respondent interactions) should be employed *with persons similar to those to be surveyed* to assess and improve all key questions along these various dimensions.” [1]

Guideline 7. Pretest questionnaires and procedures to identify problems prior to the survey.

“ … Because it is rarely possible to foresee all the potential misunderstandings or biasing effects of different questions or procedures, *it is vital for a well-designed survey operation to include provision for a pretest. All questions should be pretested to ensure that questions are understood by respondents*, can be properly administered by interviewers or rendered by web survey software and do not adversely affect survey cooperation.” [1]

**Figure 2.**
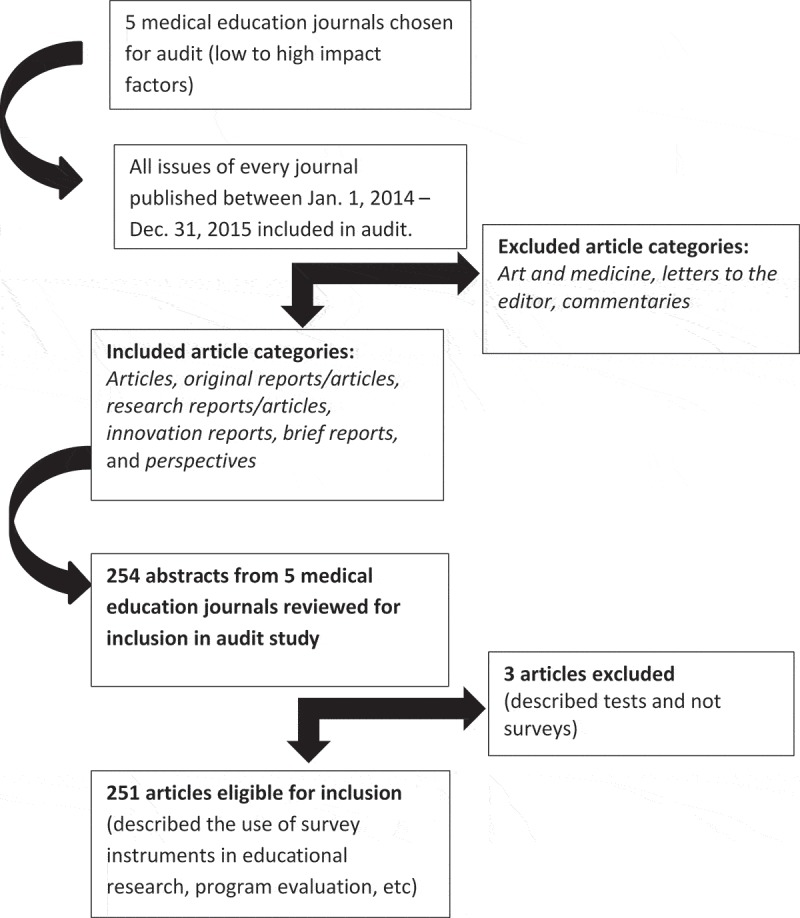
Article selection process for the audit of survey reporting practices in five journals.

#### Journal selection criteria

National and international journals indexed in PubMed which focused on undergraduate and postgraduate medical education were included. Unlike previous studies examining only journals with the highest impact factors in their fields [3,5,26,50], we purposefully selected journals with a range of impact factors (low to high). We also included journals such as the *Journal of Surgical Education*, which were ‘high-output’ survey publishers (i.e., those which published a large volume of survey articles). Though the number of journals audited was small (five), our study was similar to three studies outside medical education which performed in-depth reviews of survey reporting practices (four to five journals) [5,26,50] and an analysis of survey research in health professions education (three journals). The following journals were included in the audit study: *Academic Medicine, Journal of Graduate Medical Education, Journal of Surgical Education, Medical Education, and Perspectives on Medical Education.*

#### Audit methodology

Audit methodology is common across fields as diverse as finance [38], quality improvement [34,37], clinical care [36,51], and social science research [35]. Audits involve an examination of practices, processes, materials, or outcomes against established criteria/standards, guidelines, best practices, or laws [34–38]. Most audits are performed with a goal of bringing about changes in practice [34,36,38]. We chose this methodology as it would allow us to evaluate how pretesting practices are reported across a subset of medical education journals and then compare published descriptions of pretesting with accepted standards or best practices within the field of survey science [1,2,6,8,10–12,14,15,18,21,22,24,44,46].

#### Audit procedure

Once journals were selected, JCF and CYC identified the following article categories to be included in the audit: *articles, original reports/articles, research reports/articles, innovation reports, brief reports*, and *perspectives*. The following categories were excluded: *letters to the editor, art and medicine, commentaries*. All survey articles (rather than a sample) published within these five medical education journals for the time period January 2014 – December 2015 were eligible for inclusion. This time period was chosen to ensure articles were no longer under embargo as we began our audit in 2016.

CYC and JCF then systematically reviewed each journal’s table of contents. Next, all abstracts (except within excluded categories) within every journal issue for the time period Jan. 2014 through Dec. 2015 were reviewed for relevance. See Figure 2. Methods and results sections of all abstracts were analyzed. Examples of terms within abstracts which triggered full article access included, but were not limited to the following: *survey, surveys, surveyed, surveying, survey design/study, question(s), questionnaire(s), poll, polling, polled, probe(s), probing, verbal probing, query, queries, querying, response, respond, respondent(s), respondent debriefing, debriefing, pretest, pretesting, pre-piloting, testing items, assessing items/questions, cognitive interview(s), cognitive interviewing, cognitive testing, think aloud, beliefs, perspectives, opinions, thoughts*. In addition, if article relevance was unclear, the article was pulled and examined. Thus, the process to identify articles went beyond a typical key word search. All titles of articles pulled for review were then entered into a data extraction form, an Excel spreadsheet, by journal name.

Based upon a review of survey science literature and published recommendations from survey methodologists, CYC and JCF developed a coding protocol (Figure 1) focusing on pretesting, as this is a critical, but frequently overlooked step in survey development. The coding protocol was piloted and refined (e.g., a code for non-survey was added to the protocol) prior to use during the 2016 audit. The following codes were used in the data extraction form: 0 – Not a survey (e.g., when the tool described was actually not a survey); 1 – New instrument, no pretesting/pre-piloting reported; 2 – New instrument, pretesting/pre-piloting reported or steps described; 3 – Pre-existing instrument (or items from pre-existing instruments), no pretesting/pre-piloting reported with target population in new study/curriculum; 4 – Pre-existing instrument, pretesting/pre-piloting reported or steps described.

To be coded as a ‘2’ or ‘4’, authors merely had to mention methods recommended for pretesting survey items in the survey science literature (e.g., pretest, pretested, pretesting, pre-pilot, pre-piloted, pre-piloting, testing items/surveys, cognitive interviews/cognitive testing, survey evaluation, feedback on items, verbal probes, think a louds, etc.). We specifically searched for language within the text of each article which indicated that authors had pretested survey tools with respondents who were similar to members of the target population, as this is essential for establishing construct validity and recommended by survey methodologists and scientists [1,2,22,24,25]. For example, in cases where authors described utilizing a previously developed instrument, but did not report testing/pretesting items prior to use, the article was coded as a ‘3’ (Appendix 1). When disagreements about coding arose, CYC and JCF discussed those items and reached consensus concerning any changes to coding.

### Results

Of 254 articles initially selected for review, three described the development of tests (rather than surveys) and were omitted from the analysis. In aggregate, a total of 251 articles described the use of survey methodology. The number of survey articles published by audited journals during the study time period ranged from 11 to 106 (Median = 37) and the range of pretesting/pre-piloting reported was 0–13.51%. Additionally, 181 of 251 articles (72.11%) described the use of a new survey instrument, but failed to report survey or item pretesting prior to use (Table 1). Fifty-three of 251 articles (21.12%) described the use of a pre-existing survey instrument (or items from a pre-existing instrument), but no articles (0%) reported pretesting the survey tool prior to use. Several of these authors noted that survey instruments were validated previously or items were derived from ‘valid’ survey instruments, yet no description of pretesting with individuals similar to the *new* target population was provided (see Discussion). Lack of pretesting was also not mentioned as a limitation in the majority of articles.

Of 251 articles, only 17 (6.77%) described using a new instrument that was pretested prior to use (Table 2). What follows are examples of descriptions of pretesting from articles which were coded as ‘2’ during the audit (new instrument, pretesting/pre-piloting reported or steps described).

**Table 2.** Audit data for a sample of medical education journals publishing survey articles from 2014 to 2015.

			New instruments used		Existing instruments used	
Journals Reviewed (all indexed in PubMed)	Audience (National, International)	Number of survey articles published^a^ (of 251)	Pretesting not reported (%)	Pretesting reported^b^ (%)	Pretesting not reported (%)	Pretesting reported (%)
**Journal 1** *(Low IF)*^c^	**National**	**106**	**89 (83.96%)**	**5 (4.72%)**	**12 (11.32%)**	**0 (0.00%)**
**Journal 2** (*High IF*)	**International**	**86**	**56 (65.12%)**	**6 (6.98%)**	**24 (27.91%)**	**0 (0.00%)**
**Journal 3** *(No IF reported)*	**National**	**37**	**26 (70.37%)**	**5 (13.51%)**	**6 (16.22%)**	**0 (0.00%)**
**Journal 4** *(No IF reported)*	**International**	**11**	**5 (45.5%)**	**0 (0%)**	**6 (54.55%)**	**0 (0.00%)**
**Journal 5** *(High IF)*	**International**	**11**	**5 (45.5%)**	**1 (9.08%)**	**5 (45.45%)**	**0 (0.00%)**

^a^Number of survey methodology articles published during time 2-year period (of 251).

^b^Any description which explicitly stated testing/pretesting of surveys/survey items had occurred or described steps in pretesting.

^c^Impact factors reported on journal web sites for 2015.

Descriptions of pretesting were often brief and provided few to no details about specific pretesting methods:

“Content validation was accomplished via review by experts, including program directors. Cognitive pretesting with 4 interns who had recently selected residency programs informed minor revisions” (p. 22) [52].

“Prior to administration, the survey underwent expert review by colleagues as well as cognitive pre-testing with a group of third year medical students from our institution” (p. 750) [53].

“Before dissemination, the survey was tested amongst student representatives of the college, who completed the questionnaire and provided feedback to inform the final version”(p. 663) [54].

In some cases, authors referred readers to other articles which described the development of an instrument:

“The survey instrument was developed and tested in 2008–2009 by experts in survey research, organizational science, and academic medicine. Literature reviews, faculty focus groups, and cognitive interviews were used to inform its development” (p.356) [55].

Other articles included more detailed reports of survey development, including pretesting methodology:

“We also engaged in detailed cognitive pretesting to identify problems with the survey questions that could result in response error (e.g., complicated instructions, vague wording, inappropriate assumptions). We conducted cognitive interviews with a small number of faculty members similar to those in our target population, using a think-aloud approach and verbal probing techniques. We then modified the survey questions on the basis of our findings” (p. 302) [56].

“The survey methodology has been detailed in a prior publication. Briefly, we developed an initial pool of questions using qualitative methods and detailed interviews, which we then subjected to Dillman’s 4 stages of pretesting, including review by knowledgeable colleagues, cognitive interviewing, pilot testing, and a final check” (p. 578) [57].

“The survey was developed based on clinical reasonableness, with input from an associate programme director of the medicine residency and the medical director of the rapid response system. It underwent cognitive testing with two medical residents, and questions and response sets were revised based on their responses. Because the available population of residents is comparatively small, we did not conduct further pre-testing of the instrument in order to avoid contaminating the subject pool” (p. 1213) [58].

Overall, the majority of descriptions of pretesting did not disclose the amount of information recommended by AAPOR and other organizations, as there was often no mention of size of pretest groups and specific pretesting methods which were used (e.g., cognitive interviews, verbal probing methods, focus groups, *think a louds*, respondent debriefings, etc.).

### Discussion and implications

In this study, only 6.77% of survey articles (N = 251 articles) published within five medical education journals over a two-year period reported pretesting survey items prior to use. No audited survey articles (0%) reported pretesting an existing instrument, though surveys are frequently used within new contexts and with new target populations and thus require pretesting [2,3,22]. This audit study goes beyond findings from previous studies reported in the medical education and health professions education literature as it provides data on survey reporting practices both within and beyond research contexts in audited journals. While Artino et al.’s [3] work is important in highlighting problems in overall quality of published survey instruments, it focused exclusively on *research* surveys published within three high-impact health professions education journals in 2013 (37 self-administered surveys in 36 research articles). In this study, we audited 251 medical education articles published during 2014–15 in journals with a range of impact factors (low to high) which reported results of evaluation projects, curriculum development projects, and research studies where survey methodology had been used. We discovered that articles published within high impact factor journals did not report pretesting more frequently than articles in low impact factor journals.

Survey methodology is widely used within the field of medical education and is one of the most commonly used methodologies in health professions education research (3). It is also integral to numerous program evaluation models, including the Kirkpatrick model [59], which has been embraced by medical educators as they evaluate single programs or units within curricula. Survey methodology is also used in large-scale evaluations of programs or organizations, and data derived from surveys can influence program adoption, continuance, funding, policies [60] and politics.

While numerous factors affect the quality of data derived from survey instruments (e.g., adequacy of content coverage in the instrument, scale development and refinement, sampling, problems in implementation in the field), if different respondents do not understand questions in the same way and as researchers intended, then the other issues are moot [2,8,9,11,12,15,18,21,22,24,40,43,47,48,61,62]. Examining response processes also provides one source of evidence recommended to establish a validity argument which supports the appropriate use and interpretations of survey results [47]. Therefore, methods which focus on the question-response process are viewed as key to determining whether questions are understandable and acceptable to members of target populations [1,2,5,22,44].

Our results were somewhat surprising, given both the literature and guidelines describing the need for assessing the quality of survey items prior to use [1,2,4,18,22,23,25,46]. Yet, our results do support findings from other fields. Bennett et al. [5], in a review of the top five journals from internal medicine, health sciences, informatics and public health, found that fewer than 20% of articles (N = 117 articles) included descriptions of survey development, including pretesting. Duffett et al. [26] also found low reporting levels of survey pretesting across five journals in critical care medicine. In nephrology, Ho-Ting Li et al. [50] found 27% of survey articles within a sample of four journals mentioned gathering validity evidence related to survey development. Finally, Artino et al. [3], in an examination of 36 survey research articles published in 2013 in three high-impact journals, found that only four articles (10.8%) reported survey pretesting which involved cognitive processes.

#### Use of ‘validated’ tools

In this sample of 251 survey articles, 21.12% utilized pre-existing questionnaires or items from pre-existing questionnaires, many of which were described as ‘validated.’ While researchers often explore pre-existing questionnaires for sample questions [6], problems arise when they fail to assess survey items with their own study populations (3) prior to data collection. Just as no measurement tools contain validity, no *questions* can contain validity either [48,61,63]. Educators, researchers, and program evaluators cannot be assured that the questions they construct, adopt, or adapt will measure constructs they are interested in measuring – or that score interpretations are actually valid – if they fail to assess these questions with representative members of new target populations prior to use [1,12,15,22]. Collecting data without pretesting with a local sample can thus lead to inaccuracies in survey results [9].

#### Limitations

The paper reports the results of an audit of 251 survey articles from five medical education journals over a 2-year time period. Journals not indexed in PubMed at the time of our review were not included in this limited audit, nor were online-only journals. We also did not include clinical journals from nursing, pharmacy, or allied health education or journals which publish medical education reports on an intermittent basis [64]. There is also a possibility that survey articles coded as ‘1’ (new instrument, no pretesting) and ‘3’ (pre-existing instrument, no pretesting) had actually included survey instruments which were pretested, but the authors failed to report it (e.g., due to article space limitations) [7]. While it is possible that journal space limitations or lack of explicit journal instructions caused authors to omit pretesting descriptions, we do not believe that this affected the results of this particular study, given earlier reports in the medical education literature on widespread problems with survey development [3,7,23] and our own experiences in the field. In addition, in order to be coded as a ‘2’ (new instrument, pretested) or ‘4’ (existing instrument, pretested) in this audit study, an author simply had to note that their ‘survey was pretested’ prior to use. Finally, while we did not formally calculate interrater agreement, we did utilize a data extraction form with explicit categories and descriptors (Figure 1), and achieved consensus on coding whenever we experienced discrepancies.

## Conclusion

Survey methodology is used in large-scale evaluations of programs or organizations; medical education research, projects, and evaluations. Data derived from surveys can influence program adoption, continuance, funding, and policies [7,10,25,60]. Despite the prevalence of surveys as a common data collection method, it appears that many of the surveys used in evaluation projects and medical education research reported in articles examined in this audit study may have contained questions which were never assessed for comprehensibility, acceptability (e.g., cultural), and answerability prior to use. Findings from this audit study indicate that reported pretesting of survey tools prior to use may be even lower than that reported in healthcare studies [5,26,50].

Given the prevalence of survey methods (especially self-administered questionnaires) across health professions education [3,4] and the potential impact of findings, we believe it is imperative that evaluators, researchers, and clinician educators adhere to accepted standards of survey development by pretesting survey questions prior to use. All survey questions, even seemingly ‘objective’ or ‘simple’ items (e.g., demographic questions), should be pretested prior to use to ensure we are measuring what we think we are measuring (the construct of interest) [2,4,6,8,15,19,22,25,40,43]. While psychometric testing is important in assessing aspects of question quality and item performance, it cannot help us to understand how our respondents, based upon their own lived experiences, interpret questions we pose (i.e., *verstehen*) [65]. Therefore, some researchers are turning to mixed method approaches (quantitative and qualitative) to ‘improve the validity of survey estimates’ (p. 152) [66]. Last, we recommend that authors, reviewers, and editors involved in the publication of survey articles and reports adhere to best practices in survey methodology, which includes transparency in reporting survey methods and disseminating survey results [1,5,67].
